# Real-world safety and effectiveness of alemtuzumab as a conditioning regimen for hematopoietic stem cell transplantation

**DOI:** 10.1007/s12185-025-04033-w

**Published:** 2025-07-07

**Authors:** Yukie Sasakura, Makiko Hatanaka, Yoshinobu Kanda

**Affiliations:** 1 General Medicine Medical, Sanofi K.K., Tokyo Opera City Tower, 3-20-2 Nishishinjuku, Shinjuku, Tokyo 163-1488 Japan; 2 Post Authorization Regulatory Study, Medical Affairs, Sanofi K.K., Tokyo, Japan; 3 Division of Hematology, Department of Medicine, Jichi Medical University, Saitama, Japan

**Keywords:** Alemtuzumab, CD52, HSCT, Post-marketing surveillance

## Abstract

Alemtuzumab, a humanized monoclonal antibody directed against CD52, is indicated for administration prior to allogeneic hematopoietic stem cell transplantation (HSCT) in Japan. This post-marketing surveillance study was conducted as part of a risk management plan to confirm the safety and effectiveness of alemtuzumab in clinical practice, as mandated by the Japanese health authorities. Fifty-nine patients aged 0 to 69 years received alemtuzumab prior to HSCT for hematologic malignancies (n = 22), aplastic anemia (n = 7), or other diseases (n = 30). The number of mismatched human leukocyte antigens between donor and recipient was ≥ 2 in 39 patients (66.1%), 1 in seven patients (11.9%) and 0 in 11 patients (18.6%). Overall, 38 of 59 patients (64.4%) developed an adverse drug reaction (ADR), most commonly fever associated with infusion reactions (n = 22); 24 patients (40.7%) had a serious ADR (most commonly cytomegalovirus-related events [n = 7]), and 16 had a grade ≥ 3 ADR (mainly febrile neutropenia [n = 3]). Engraftment was achieved in 55 of 58 patients (94.8%) at a median of 16 days after HSCT, and predefined success (engraftment without grade ≥ 3 graft-versus-host disease in hematologic malignancies or grade ≥ 2 in other diseases) was met in 51 of 58 patients (87.9%). These results support the manageable safety profile and effectiveness of alemtuzumab as preconditioning for HSCT in Japanese patients.

**Supplementary Information:**

The online version contains supplementary material available at 10.1007/s12185-025-04033-w.

## Introduction

Hematopoietic stem cell transplantation (HSCT) is an established approach to the treatment of a range of hematologic malignancies, non-malignant immune disorders and high-risk autoimmune diseases [1]. Acute graft-versus-host disease (GVHD) is one of the major complications of allogeneic HSCT, and a leading cause of early transplant-related mortality, particularly when there are human leukocyte antigen (HLA) mismatches between donor and recipient [2, 3]. Over the last several decades, considerable research has been undertaken to optimize conditioning regimens and prophylaxis protocols for HSCT in order to achieve sufficient myelotoxicity for transplant success while minimizing the risk of infections, GVHD and transplant-related mortality [4].

Alemtuzumab is a humanized monoclonal antibody directed against CD52, an antigen that is expressed on more than 95% of peripheral blood lymphocytes, monocytes, eosinophils and macrophages, but not on erythrocytes, platelets, granulocytes or hematopoietic progenitor cells [5]. When used to deplete T cells prior to HSCT, alemtuzumab has been shown to reduce the incidence of severe acute GVHD among patients receiving HSCT with excellent disease-free survival, but with a high rate of viral reactivations caused by delayed immune cell recovery [6–13].

The effectiveness and safety of alemtuzumab at preventing acute GVHD in haploidentical HSCT have been demonstrated in one Japanese clinical study involving patients with hematologic malignancies undergoing HSCT using grafts from donors with two or three HLA mismatches (n = 12) [14] and in two Japanese investigator-initiated clinical trials; one (HE0402) trial in patients with relapsed/refractory acute leukemia undergoing haploidentical HSCT using grafts from donors with two or three HLA mismatches (n = 14) and the other (HE0403) in patients with aplastic anemia (n = 15) [15]. Alemtuzumab was approved as a conditioning regimen for HSCT in Japan in December 2020 [16, 17]. Herein, we describe the results of post-marketing surveillance (PMS), which was conducted as mandated by the Japanese health authorities as part of a risk management plan to confirm the safety and effectiveness of alemtuzumab as a preconditioning regimen in Japanese patients undergoing HSCT in real-world clinical practice.

## Materials/subjects and methods

### Study design

This was an observational study conducted at 20 hospitals in Japan. Investigators at these centers prospectively enrolled all patients who received alemtuzumab prior to allogeneic HSCT between March 22, 2021, and March 31, 2023. Patients who had received alemtuzumab after its approval in Japan in December 2020 were also included retrospectively. The observation period was from the date of the first alemtuzumab infusion to 60 days after the HSCT, or until the last follow-up. Investigators completed questionnaires and case report forms detailing patient characteristics, medical history, treatment received, donor information, concomitant medications, outcomes and the occurrence of any adverse events (AEs). Data were based on HLA mismatches at the antigen level.

The approved dose of alemtuzumab as a conditioning regimen for HSCT in Japan is 0.16 mg/kg administered by intravenous (IV) infusion once daily for 6 days. Treatment modifications in this PMS were based on the discretion of the treating physicians.

### Outcomes

Effectiveness was evaluated by treatment success rate, engraftment status, survival rate and the proportion of individuals without acute GVHD. Treatment success was defined as the proportion of patients who survived for ≥ 60 days and achieved engraftment after HSCT, and did not develop acute GVHD of grade ≥ 3 (for patients with hematologic malignancies) or grade ≥ 2 (for patients with other diagnoses) severity [15]. Engraftment was defined as a peripheral blood neutrophil count of > 500 cells/mm^3^ for 3 consecutive days in the first 60 days post-HSCT (the first date on which this threshold was met was used to define the date of engraftment) [18]. GVHD severity was rated according to the National Cancer Institute Common Terminology Criteria for Adverse Events (NCI CTCAE) version 5.0.

Safety was evaluated by the occurrence of AEs within 60 days of HSCT. AEs were classified by system organ class and preferred term according to the Japanese version of the Medical Dictionary of Regulatory Affairs (MedDRA/J) version 26.0. All AEs were evaluated for severity (using NCI CTCAE version 5.0), seriousness (see Supplementary methods for definitions of serious AEs), possible causal relationship to alemtuzumab and outcome. AEs considered to be related to alemtuzumab were classified as adverse drug reactions (ADRs). The AEs of special interest (i.e., listed in the safety specification for alemtuzumab) were infusion reactions, infections, hematotoxicity, autoimmune hemolytic anemia, autoimmune thrombocytopenia, hemorrhage, cardiac disorder, progressive multifocal leukoencephalopathy (PML) and reactivation of hepatitis B virus.

### Ethical considerations

The study was conducted in compliance with the Ministerial Ordinance on Standards for Good Post-marketing Study Practice (GPSP) in Japan. Patients consented to treatment, as per usual clinical practice. Under GPSP regulations, patients had the opportunity to discontinue their participation in the research but explicit written informed consent for the use of their data was not required.

### Statistical analysis

Effectiveness and safety outcomes were evaluated using descriptive statistics. Categorical variables were summarized using the number and proportion of patients, and continuous variables were analyzed by the mean and standard deviation (SD), median and range. For subgroup comparisons, the Fisher’s exact test was primarily used, but the Cochran–Armitage test was used when the relevant variable was measured on an ordinal scale with ≥ 3 categories. The engraftment rate over time was evaluated using Kaplan–Meier method, with 95% confidence intervals (CI) also calculated. All tests were performed at a two-sided significance level of 5%. No imputation was made for missing data.

## Results

### Patients

A total of 59 patients were enrolled at 20 centers across Japan and evaluated for safety; one patient was not evaluable for effectiveness, so effectiveness was analyzed in 58 patients (see Supplementary results for further information on the effectiveness analysis set). Of all 59 patients, 39 were males (66.1%), 20 were females (33.9%), and the median age was 22 years (< 15 years, n = 22 (37.3%); 15–64 years, n = 36 (61.0%); ≥ 65 years, n = 1 (1.7%) (Table 1).

**Table 1:** Participant characteristics

	Safety analysis set (n = 59)
Male, n (%)	39 (66.1)
Age, mean ± SD, years	26.1 ± 20.3
Age categories, n (%)	
< 15 years	22 (37.3)
≥ 15 to < 65 years	36 (61.0)
≥ 65 years	1 (1.7)
Bodyweight, mean ± SD, kg	44.2 ± 20.4
Primary disease, n (%)	
Hematologic malignancy	22 (37.3)
Aplastic anemia	7 (11.9)
Other	30 (50.9)
Number of HLA mismatches, n (%)	
No mismatch	11 (18.6)
Single-locus mismatch	7 (11.9)
≥ 2 loci mismatches	39 (66.1)
Not reported	2 (3.4)
Comorbidities^a^, n (%)	20 (33.9)
Infectious disease	11 (8.6)
Liver disease	6 (10.2)
Other	8 (13.6)
Complications^a^, n (%)	28 (47.5)
Infectious disease	9 (15.3)
Liver disease	6 (10.2)
Cardiac disease	3 (5.1)
Kidney disease	1 (1.7)
Other	20 (33.9)
Pre-HSCT treatment, n (%)	
Drug therapy (other than alemtuzumab)	54 (91.5)
Radiation	25 (42.4)
Other	1 (1.7)
Stem cell source, n (%)	
Bone marrow	22 (37.3)
Peripheral blood	36 (61.0)
Umbilical cord blood	1 (1.7)
Donor, n (%)	
Sibling	22 (37.3)
Related (non-sibling)	21 (35.6)
Unrelated	16 (27.1)

^a^Patients could have more than one comorbidity or complication

*HLA* human leukocyte antigen, *HSCT* haematopoietic stem cell transplantation, *SD* standard deviation

Primary diseases requiring HSCT were hematologic malignancies (n = 22, 37.3%), aplastic anemia (n = 7, 11.9%) and other diseases (n = 30, 50.9%; Table 1). The most common hematologic malignancies were acute myeloid leukemia (n = 7), acute lymphocytic leukemia (n = 6) and myelodysplastic syndrome (n = 3). Of other diseases, the most common were X-linked lymphoproliferative syndrome (n = 8) and chronic active Epstein–Barr virus disease (n = 7) (Table S1).

HLA type mismatches between donor and recipient were reported in 57/59 patients; 11 patients (18.6%) had a match with the donor, seven patients (11.9%) had one locus mismatch and 39 patients (66.1%) had ≥ 2 mismatched loci.

Twenty patients (33.9%) had a medical history of comorbidities, and 28 (47.5%) had complications, including infection (n = 9; 15.3%), liver disease (n = 6; 10.2%), cardiac disease (n = 3; 5.1%) or kidney disease (n = 1; 1.7%).

### Alemtuzumab and HSCT

Prior to HSCT, 54 patients (91.5%) received conditioning with a drug other than alemtuzumab, 25 (42.4%) received radiation, and one patient (1.7%) received the HLH-94 treatment protocol for hemophagocytic lymphohistiocytosis. The most common conditioning regimens were busulfan plus fludarabine phosphate (n = 15), cyclophosphamide hydrate plus total body irradiation ([TBI]; n = 7), melphalan plus fludarabine phosphate plus TBI (n = 7), busulfan plus melphalan plus fludarabine phosphate (n = 6) and busulfan plus fludarabine phosphate plus TBI (n = 5).

Stem cells for transplantation were sourced from peripheral blood in 36 patients (61.0%), bone marrow in 22 patients (37.3%) and umbilical cord blood in one patient (1.7%). Donors were siblings in 22 cases (37.3%), non-sibling relatives in 21 cases (35.6%) and unrelated individuals in 16 cases (27.1%).

Of the 59 included patients, 37 discontinued treatment during the 6-day treatment period: 30/37 (81.1%) terminated treatment as planned; 1/37 (2.7%) discontinued treatment due to primary disease progression; and 6/37 (16.2%) discontinued for other reasons (Table S2). Overall, the mean ± SD duration of treatment with alemtuzumab was 3.7 (1.1) days. Alemtuzumab was administered consecutively for 2 days in four patients (6.8%), for 3 days in 31 patients (52.5%), for 4 days in six patients (10.2%), for 5 days in 15 patients (25.4%), and for 6 days in two patients (3.4%). One patient (1.7%) received 2 courses of alemtuzumab first for 4 days before HSCT, then after an interval of approximately 40 days for another 3 days before undergoing a second HSCT. The total dosage of alemtuzumab ranged from 0.25 to 1.12 mg/kg (median 0.48 mg/kg; mean ± SD, 0.60 ± 0.19 mg/kg).

All 59 patients received concomitant medications, most commonly consisting of corticosteroids, antivirals, antihistamines, antipyretics, analgesic/anti-inflammatory agents and antibiotics. GVHD prophylaxis consisted mainly of cyclosporine and methotrexate. Alkylating agents were used as concomitant drugs for primary diseases. GVHD was confirmed in four of 58 patients included in the effectiveness analysis; a 46-year-old male (grade 3, onset on day 21, duration of 46 days), a 16-year-old male (grade 3, onset on day 39, duration of 77 days), a 37-year-old male (grade 3, onset on day 31, duration of 50 days) and an 11-year-old male (grade 2, onset on day 21, duration of 56 days).

### Safety

Overall, 38 out of 59 patients exposed to alemtuzumab (64.4%) developed an ADR (Table 2). The most common ADRs were fever associated with infusion reactions (n = 22; 37.3%), CMV-related AEs (n = 7, 11.9%), infusion-related reaction (n = 3; 5.1%), adenovirus infection (n = 2; 3.4%), nausea (n = 2; 3.4%), hemorrhagic cystitis (n = 2; 3.4%), decreased lymphocyte count (n = 2; 3.4%) and engraftment failure (n = 2; 3.4%).

**Table 2:** Adverse drug reactions in the safety analysis set (n = 59): Overall, by organ system class and specific events occurring in ≥ 2 patients

ADRs, n (%) SOC PT	Any	Serious	Grade ≥ 3
ADRs of any type	38 (64.4)	24 (40.7)	16 (27.1)
Infections	15 (25.4)	15 (25.4)	6 (10.2)
CMV viremia	4 (6.8)	4 (6.8)	0
CMV reactivation	3 (5.1)	3 (5.1)	0
Adenovirus infection	2 (3.4)	2 (3.4)	2 (3.4)
Blood and lymphatic system disorders	4 (6.8)	4 (6.8)	3 (5.1)
Febrile neutropenia	3 (5.1)	3 (5.1)	3 (5.1)
Immune system disorders	3 (5.1)	3 (5.1)	1 (1.7)
Gastrointestinal disorders	3 (5.1)	0	0
Nausea	2 (3.4)	0	0
Renal and urinary tract disorders	2 (3.4)	2 (3.4)	1 (1.7)
Hemorrhagic cystitis	2 (3.4)	2 (3.4)	1 (1.7)
General disorders and administration site conditions	23 (39.0)	1 (1.7)	2 (3.4)
Fever	22 (37.3)	0	2 (3.4)
Laboratory abnormalities	5 (8.5)	2 (3.4)	3 (5.1)
Decreased lymphocyte count	2 (3.4)	1 (1.7)	2 (3.4)
Injury, poisoning or procedural complications	5 (8.5)	3 (5.1)	2 (3.4)
Infusion-related reaction	3 (5.1)	1 (1.7)	2 (3.4)
Engraftment failure	2 (3.4)	2 (3.4)	2 (3.4)

*ADRs* adverse drug reactions, *CMV* cytomegalovirus, *PT* preferred term; *SOC* system organ class

Serious ADRs occurred in 24 patients (40.7%), most commonly CMV-related AEs (n = 7, 11.9%), febrile neutropenia (n = 3; 5.1%) and adenovirus infection, hemorrhagic cystitis and engraftment failure (n = 2 each; 3.4%). One 3-year-old patient died of a serious ADR (multi-organ dysfunction syndrome and pulmonary hemorrhage) 58 days after the end of alemtuzumab treatment, which he received for 4 days as planned.

At the time of database lock, four serious ADRs had not resolved (adenovirus infection, cytokine storm, vascular disorder and multiple organ dysfunction syndrome) and the outcome of one ADR (disseminated intravascular coagulation and thrombotic microangiopathy) was unknown. Apart from these four SAEs and the death reported above, all other serious ADRs were recovered or recovering.

ADRs of grade ≥ 3 occurred in 16 patients (27.1%): febrile neutropenia in three patients (5.1%); adenovirus infection, fever, decreased lymphocyte count, infusion-related reaction and engraftment failure in two patients each (3.4%); bronchopulmonary aspergillosis, CMV chorioretinitis, streptococcal bacteremia, pseudomonas sepsis and human herpesvirus-6 encephalitis, GVHD, hypoxia, hemorrhagic cystitis and hepatic enzymatic abnormalities in one patient each (1.7%).

Infusion reactions occurred in 24 patients per physician reports and in 25 patients (42.37%) per MeDRA/J AE reports. There were no cases of autoimmune hemolytic anemia, autoimmune thrombocytopenia, progressive multifocal leukoencephalopathy, or reactivation of hepatitis B virus.

There were statistically significant differences in the incidence of ADRs according to primary disease and stem cell source, and number of HLA mismatches (Table S3). Fever occurred more frequently in patients with aplastic anemia (5/7; 71.4%) or other primary diseases (13/30; 43.3%) than in those with hematologic malignancies (4/22; 18.2%).

### Effectiveness

**Fig. 1:**
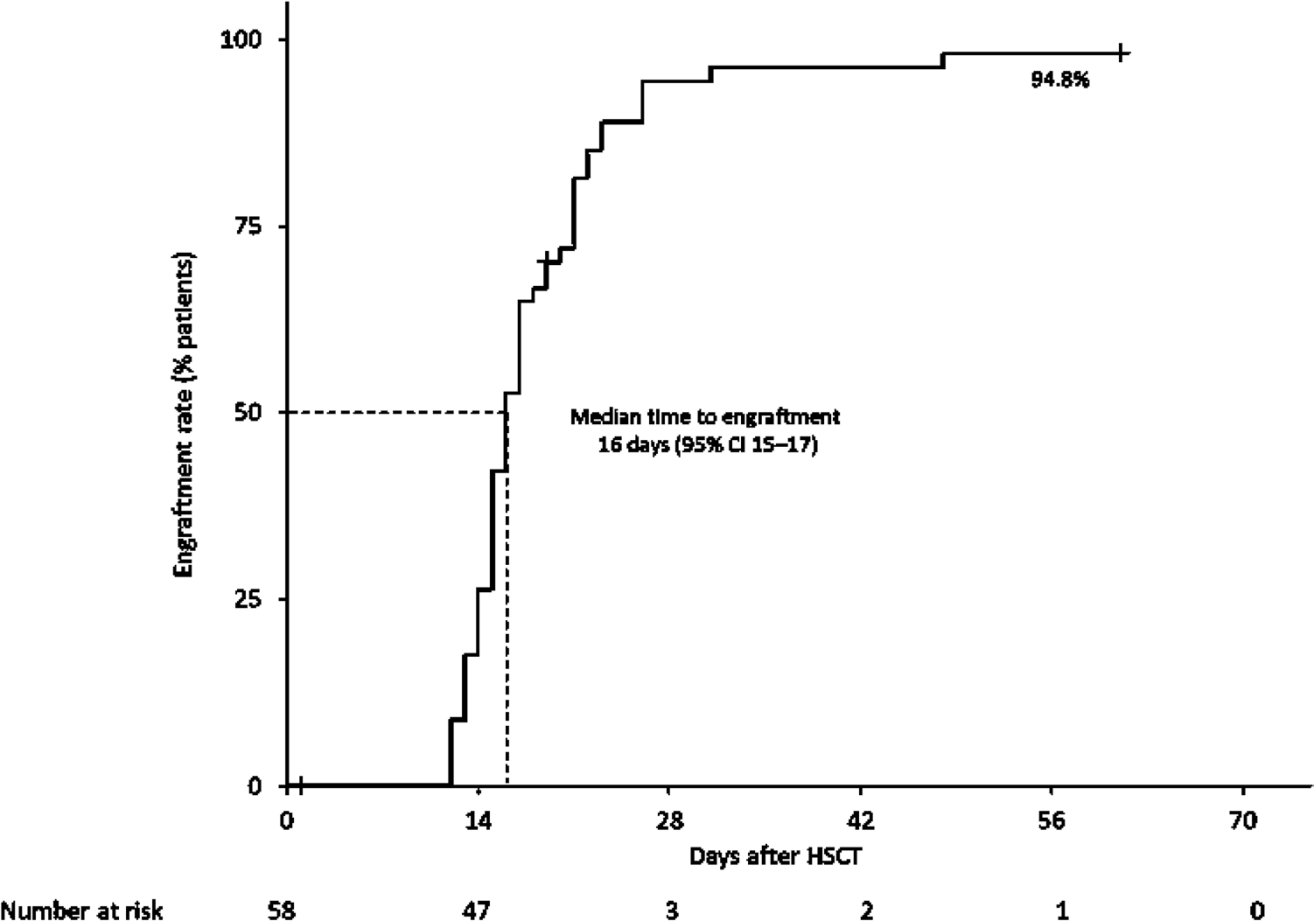
Kaplan–Meier graph for time to engraftment in the effectiveness analysis set (n = 58). *CI* confidence interval, *HSCT* hematopoietic stem cell transplant

Of 58 patients evaluable for effectiveness, 55 achieved engraftment between 12 and 48 days (median 16; 95% CI 15–17) after HSCT (Fig. 1); therefore, the engraftment rate was 94.8% (95% CI 85.6 to 98.2). Three patients (one child [described earlier] and two adults) died within 60 days of undergoing HSCT, therefore the overall survival rate was 94.8% at day 60. The child (3 years; male) was diagnosed with thrombotic thrombocytopenic purpura and died 49 days after HSCT. An 18-year-old female patient who had posterior reversible encephalopathy syndrome died 28 days after HSCT and a 58-year-old male patient who had acute respiratory distress syndrome died 19 days after HSCT. Although we did not collect information about early death, this possibility cannot be denied. Engraftment was confirmed in all three cases before death. Therefore, these deaths were deemed to not be related to delayed engraftment.

Acute GVHD developed in four patients, including grade ≥ 3 acute GVHD in one patient with hematologic malignancies and grade ≥ 2 acute GVHD in three patients with other diseases; therefore, the success rate was 87.9% (n = 51/58; Table 3). The success rate was 90.5% in children aged < 15 years (n = 19/21), 86.5% in adults aged ≥ 15 to 64 years (n = 32/37) and 100.0% in an older patient aged 65 years who met the criteria for success.

**Table 3:** Treatment response by total dose in the effectiveness analysis set (n = 58)

Total dose (mg/kg/day)	n	Engraftment, n		Acute GVHD, n		Success^a^	
		Yes	No	Yes	No	n	%
< 0.2	0	0	0	0	0	–	–
≥ 0.2 to < 0.4	4	4	0	0	4	4	100.0
≥ 0.4 to < 0.6	29	28	1	2	27	26	89.7
≥ 0.6 to < 0.8	7	7	0	1	6	6	85.7
≥ 0.8 to < 1.0	18	16	2	1	17	15	83.3
Total	58	55	3	4	54	51	87.9

^a^Success was defined as engraftment and no acute GVHD of grade ≥ 2 or ≥ 3

*GVHD* graft-versus-host disease

## Discussion

This mandatory PMS among Japanese patients receiving alemtuzumab as a conditioning regimen for HSCT supports the manageable safety profile and effectiveness of alemtuzumab in this setting, consistent with previous reports. No new safety concerns were identified.

While large-scale retrospective studies of alemtuzumab have been conducted in Europe [19, 20], previous reports on the safety and efficacy of this drug in Japanese patients are based on small studies including only 12–15 patients [14, 15], so this is the first report assessing safety and effectiveness of alemtuzumab as a conditioning regimen in a large cohort of Japanese patients. The incidence of ADRs in our study (64.4%) was lower than the incidence reported in the two investigator-initiated clinical trials in Japanese individuals with hematologic malignancies (HE0402) or aplastic anemia (HE0403) (Table S4) [15].

Our study included an unselected population of children and adult patients exposed to alemtuzumab conditioning for a wide range of malignant and nonmalignant diseases. The opposite was true for the highly selected patients enrolled in previous Japanese studies, who had to satisfy an Eastern Cooperative Oncology Group (ECOG) performance status of < 2 (both HE0402 and HE0403 studies), had to have at least two HLA antigen mismatches (HE0402 study only), and their underlying diseases were less heterogeneous than those in our study population [15]. However, reporting of AEs in clinical practice may be less accurate than in clinical trials, which are usually closely monitored [21].

The types of ADRs seen in the current study and previous Japanese studies with alemtuzumab were comparable, being mainly infectious ADRs, such as CMV activation and adenovirus infection [15]. These ADRs may be explained by the duration of effective blood concentrations of alemtuzumab, which persist long after the treatment course has ended. In Japanese adults, alemtuzumab has a half-life of ~ 10 days [15]. Serum levels of alemtuzumab may persist for up to 56 days after HSCT in adults [22] and for 36 days in children [23]. High levels of alemtuzumab exposure correlate with delays in CD4 + and CD8 + T cell recovery [23], which in turn increases the risk of CMV and adenovirus infections [24, 25]. Antiviral prophylaxis or pre-emptive therapy, as well as adequate patient monitoring, is recommended during alemtuzumab and after HSCT to reduce the risk of such infections [25].

In our study, fever associated with infusion reactions was the most common ADR of alemtuzumab; in 37.3% of patients overall, in 18.2% of patients with hematologic malignancies, 71.4% of patients with aplastic anemia and in 43.3% of patients with other primary diseases. In the early investigator-initiated studies, 58.3% of patients undergoing HSCT for aplastic anemia and 90.9% of patients undergoing HSCT from related donors with ≥ 2 mismatched HLA loci developed fever (Sanofi internal data). Flu-like symptoms, including fever, are common in patients receiving alemtuzumab, particularly after the first dose, but generally diminish with subsequent doses [26]. The mechanism of first-dose fever may be related to cytokine release [26], but fever can also be a sign of infection, so vigilance is required. The Japanese prescribing information for alemtuzumab contains a warning about pyrexia and a precaution about CMV viraemia [27].

The high engraftment rate (94.8%) and the rather low rate of grade 1–4 acute GVHD (20.3%) in the current study is almost consistent with previous reports in Japanese patients. In the HE402 and HE 403 studies, 100% of patients who received alemtuzumab achieved engraftment and 24.1% developed grade 1–4 acute GVHD [15]. In a recent prospective study that included 14 Japanese patients with hematologic malignancies undergoing haploidentical HSCT, 92.9% achieved engraftment, 28.6% developed grade 1 acute GVHD and 21.4% developed limited chronic GVHD [28]. This is in contrast to the results of a large-scale retrospective single-center study of 201 UK-based patients who received alemtuzumab-based reduced-intensity conditioned allogeneic HSCT, where 51.2% of patients developed grade 1–4 acute GVHD and 10.0% developed chronic GVHD [19]. In the same study, 10 mg/day of alemtuzumab was administered for 5 days (days −5 to −1) before HSCT, and most patients received GVHD prophylaxis with cyclosporin A alone. In the HE402 and HE403 Japanese studies, alemtuzumab was given at 0.16 mg/kg/day for 6 days (from days −8 to −3) and prophylaxis against GVHD was performed with cyclosporin A and short-term methotrexate [15]. In the transplant cohort reported by Kako and colleagues, alemtuzumab was given at 0.25 mg/kg/day for only 2 days (days −4 and −3 before HSCT) with a capping dose at 30 mg, and prophylaxis against GVHD was also performed with cyclosporin A and short-term methotrexate [28]. In our study, the duration of alemtuzumab treatment was left to the discretion of the physician and most patients received alemtuzumab for only 3 days, while GVHD prophylaxis was with cyclosporin A and methotrexate.

Several limitations must be considered when interpreting the results of this PMS, which are inherent of these types of studies, including the fact that only data that were available in the patients’ medical records were collected. In addition, HSCT was performed as a part of various treatment strategies, with varying doses of alemtuzumab and in patients with varying characteristics. Taken together with the different clinical situations described herein, the results of this PMS should be interpreted with caution.

## Conclusions

The current PMS supports the manageable safety profile and effectiveness of alemtuzumab as a conditioning regimen for HSCT in routine clinical practice. It showed a high engraftment rate (94.8%) and a rate of 20.3% for grade 1–4 acute GVHD, consistent with previous reports in Japanese patients. Although ADR reporting may have been underestimated due to the observational design of the study, the spectrum of ADRs was comparable with previous clinical trials involving Japanese patients, and no new safety concerns were identified. Warnings and precautions in the current Japanese product insert remain appropriate, and physicians using alemtuzumab in patients undergoing allogeneic HSCT should pay careful attention to minimize the risk of infectious complications. Further studies of alemtuzumab use as a conditioning regimen in real-world practice are warranted to verify the results of this PMS.

## Supplementary Information

Below is the link to the electronic supplementary material.

Supplementary file1 (DOCX 54 KB)

## Data Availability

This post-marketing surveillance was conducted under the Japanese Ministerial Ordinance on Good Post-marketing Study Practice for Drugs, and because of the characteristics of the surveillance in the regulation, the scope of permission for data sharing is limited to the content described in the paper; however, any inquiries should be directed to the corresponding author.
