# Supplementary material for: Real-world safety and effectiveness of alemtuzumab as a conditioning regimen for hematopoietic stem cell transplantation
Source: Int J Hematol. 2025 Jul 7;122(5):709–16. doi: 10.1007/s12185-025-04033-w (PMC12572087; doi:10.1007/s12185-025-04033-w)
Supplement: Supplementary file 1 — Supplementary file1 (DOCX 54 KB) [file 12185_2025_4033_MOESM1_ESM.docx]

**Original article**

**Real-World Safety and Effectiveness of Alemtuzumab as a Conditioning Regimen For Hematopoietic Stem Cell Transplantation**

**Authors:** Yukie Sasakura^1^ Makiko Hatanaka^2^ Yoshinobu Kanda, MD, PhD^3^

**Affiliations:** ^1^General Medicine Medical, Sanofi K.K., Tokyo, Japan; ^2^ Post Authorization Regulatory Study, Medical Affairs, Sanofi K.K., Tokyo, Japan; ^3^Division of Hematology, Department of Medicine, Jichi Medical University, Saitama, Japan

**Corresponding author:** Yukie Sasakura, Sanofi K.K., Tokyo Opera City Tower, 3-20-2 Nishishinjuku, Shinjuku, Tokyo 163-1488, Japan. Phone: +81-80-2487-4857; Fax: +81-3-6301-3200; Email: yukie.sasakura@sanofi.com

# supplementary materials

# supplementary methods

## Definition of serious adverse events

An adverse event (AE) was considered serious, as judged by the attending physician, if it met any of the following criteria:

1. Resulted in death
2. Was life-threatening
3. Required hospitalization or prolongation of hospitalization for treatment
4. Resulted in permanent or significant disability or insufficiency
5. Resulted in birth defects or birth defects
6. Resulted in other medically important conditions

# supplementary results

## Effectiveness analysis set

Of the 59 individuals enrolled in this study, one participant could not be evaluated for effectiveness. This patient received 4 days of alemtuzumab followed by hematopoietic stem cell transplantation (HSCT), and then received another 3 days of alemtuzumab followed by another HSCT approximately 40 days later. Although there were 2 cycles of treatment followed by transplantation, only the second evaluation was used in the effectiveness evaluation. Therefore, this patient was excluded from the effectiveness analysis because it was judged that an appropriate effectiveness evaluation was not obtained.

# supplementary tables

## Table S1.

Primary Diseases of Study Patients

| **Primary disease, n (%)** | **Safety analysis set (n=59)** |
| --- | --- |
| Hematologic malignancy | 22 (37.3) |
| AML | 7 (11.9) |
| ALL | 6(10.2) |
| Myelodysplastic syndromes | 3 (5.1) |
| CML blast crisis | 1 (1.7) |
| Myeloproliferative neoplasms | 1 (1.7) |
| Non-Hodgkin lymphoma | 3 (5.1) |
| Aplastic anemia | 7 (11.9) |
| Other | 30 (50.9) |
| X-linked lymphoproliferative syndrome | 8 (13.6) |
| Chronic active Epstein-Barr virus infection | 7 (11.9) |
| Chronic granulomatous disease | 3 (5.1) |
| Activated PI3-kinase δ syndrome | 2 (3.4) |
| Immunodeficiency | 2 (3.4) |
| Adult TCL/T-cell leukaemia | 1 (1.7) |
| Combined immunodeficiency | 1 (1.7) |
| Familial HLH | 1 (1.7) |
| HLH | 1 (1.7) |
| Hereditary stomatocytosis | 1 (1.7) |
| Leukocyte adhesion deficiency type 1 | 1 (1.7) |
| Primary immunodeficiency syndrome | 1 (1.7) |
| Wiskot-Aldrich syndrome | 1 (1.7) |

ALL, acute lymphocytic leukemia; AML, acute myeloid leukemia; CML, chronic myeloid leukemia; HLH, hemophagocytic lymphohistiocytosis; TCL, T-cell lymphoma.

## Table S2.

Discontinuations During Alemtuzumab Treatment in the Safety Analysis Set (n=59)

| **Treatment duration** | **n** | **Discontinuations, n**^a^ | **Reasons for discontinuation, n (%)** | | |
| --- | --- | --- | --- | --- | --- |
|  |  |  | **Terminated as planned** | **Primary disease progression** | **Other reason^c^** |
| 1 day | 0 | 0 | 0 | 0 | 0 |
| 2 days | 4 | 3 | 2 (66.7) | 0 | 1 (33.3) |
| 3 days | 31 | 14 | 11 (78.6) | 0 | 3 (21.4) |
| 4 days | 5 | 3 | 3 (100.0) | 0 | 0 |
| 5 days | 15 | 14 | 13 (92.9) | 0 | 1 (7.1) |
| 6 days | 2 | 1 | 0 | 1 | 0 |
| Other^b^ | 2 | 2 | 1 (50.0) | 0 | 1 (50.0) |
| Total | 59 | 37 | 30 (81.1) | 1 (2.7) | 6 (16.2) |

^a^Discontinuations due to adverse events, not presenting for treatment, or patient request.

^b^Both participants received alemtuzumab twice. The first patient received alemtuzumab for 4 days, underwent HSCT, then after an interval of approximately 40 days received alemtuzumab for another 3 days before undergoing a second HSCT. The second patient received alemtuzumab for 3 days, then after an interval of 4 days received alemtuzumab again before undergoing HSCT.

^c^Two patients discontinued alemtuzumab to avoid adverse events (treatment durations were 3 and 5 days, respectively), and two patients discontinued due to transplantation pretreatment (treatment durations were 2 and 3 days, respectively). One patient discontinued because the alemtuzumab dose appeared to be sufficient after 3 days of treatment, and one patient did not receive additional alemtuzumab doses after 3 continuous days (the total treatment duration was 8 days).

HSCT, hematopoietic stem cell transplant.

## Table S3.

Incidence of Adverse Drug Reactions According to Patient Characteristics in the Safety Analysis Set (n=59)

|  | **N** | **ADRs, n (%)** | **P-value** |
| --- | --- | --- | --- |
| Sex | | | |
| Male | 39 | 25 (64.1) | 1.00^a^ |
| Female | 20 | 13 (65.0) |  |
| Age category | | | |
| <15 years | 22 | 16 (72.7) | 0.247^a^ |
| 15 to 64 years | 36 | 22 (61.1) |  |
| ≥65 years | 1 | 0 |  |
| Primary disease | | | |
| Hematologic malignancy | 22 | 9 (40.9) | 0.014^a^ |
| Aplastic anemia | 7 | 6 (85.7) |  |
| Other | 30 | 23 (76.7) |  |
| HLA compatibility classification | | | |
| Match | 11 | 9 (81.8) | 0.025^a^ |
| One locus mismatch | 7 | 7 (100.0) |  |
| Two or more loci mismatch | 39 | 20 (51.3) |  |
| Not evaluable | 2 | 2 (100.0) |  |
| Stem cell source | | | |
| Bone marrow | 22 | 18 (81.8) | 0.025^a^ |
| Peripheral blood | 36 | 20 (55.6) |  |
| Umbilical cord blood | 1 | 0 |  |
| Complications | | | |
| None | 31 | 17 (54.8) | 0.173^a^ |
| Present | 28 | 21 (75.0) |  |
| Donor | | | |
| Sibling | 22 | 15 (68.2) | 0.098^a^ |
| Non-sibling relative | 21 | 10 (47.6) |  |
| Unrelated | 16 | 13 (81.3) |  |
| Total dose (mg/kg/day) | | | |
| ≥0.2 to <0.4 | 4 | 3(75.0) | 0.795^b^ |
| ≥0.4 to <0.6 | 29 | 17(58.6) |  |
| ≥0.6 to <0.8 | 7 | 7(100) |  |
| ≥0.8 to <1.0 | 18 | 11(61.1) |  |
| ≥1.0 | 1 | 0(0) |  |

^a^Fisher’s exact test.

^b^Cochran–Armitage test.

ADRs, adverse drug reactions; HLA, human leukocyte antigen.

## Table S4.

Incidence of Adverse Events in the Previous Studies in Japanese Patients (Kanda 2013) and in the Current Post-Marketing Drug-Use Results Survey

|  | **Study HE0402 (n=14) and HE0403 (n=15)** | **Patients in the Current Survey (n=59)** | |
| --- | --- | --- | --- |
|  | **AEs, % (95% CI)** | **AEs, %** | **ADRs, %** |
| Infections | 75.9 (64.0–87.7) | 35.6 | 25.4 |
| Hematologic toxicity | 58.6 (45.0–72.3) | 23.7 | 8.5 |
| Bleeding | 58.6 (45.0–72.3) | 6.8 | 6.8 |
| Heart disorders | 41.4 (27.7–55.0) | 3.4 | 1.7 |

ADRs, adverse drug reactions; AEs, adverse events; CI, confidence interval.
